# Spread of Carbapenem-Resistant Klebsiella pneumoniae Clinical Isolates Producing NDM-Type Metallo-β-Lactamase in Myanmar

**DOI:** 10.1128/spectrum.00673-22

**Published:** 2022-06-28

**Authors:** Satomi Takei, Yu Jie Lu, Mari Tohya, Shin Watanabe, Shigeki Misawa, Yoko Tabe, Takashi Miida, San Mya, Htay Htay Tin, Tatsuya Tada, Teruo Kirikae

**Affiliations:** a Department of Clinical Laboratory, Juntendo University grid.258269.2grid.411966.dgrid.258269.2 Hospital, Tokyo, Japan; b Department of Clinical Laboratory Medicine, Juntendo University grid.258269.2grid.411966.dgrid.258269.2 Graduate School of Medicine, Tokyo, Japan; c Department of Microbiology, Juntendo University grid.258269.2grid.411966.dgrid.258269.2 Graduate School of Medicine, Tokyo, Japan; d Department of Microbiome Research, Juntendo University grid.258269.2grid.411966.dgrid.258269.2 Graduate School of Medicine, Tokyo, Japan; e National Health Laboratory, Yangon, Myanmar; Instituto Oswaldo Cruz

**Keywords:** carbapenemase-producing *Enterobacteriaceae*, *Klebsiella pneumoniae*, NDM-type metallo-β-lactamase, 16S rRNA methylase

## Abstract

A total of 38 isolates of carbapenem-resistant Klebsiella pneumoniae harboring *bla*_NDM_ were obtained during surveillance of 10 hospitals in Myanmar. Of these 38 isolates, 19 (50%) harbored genes encoding 16S rRNA methylases, such as *armA* or *rmtB*. The K. pneumoniae strains tested belonged to 17 sequence types (STs), including the high-risk clonal lineages ST101 and ST147. The ST101 and ST147 isolates carried IncFII plasmids harboring *bla*_NDM-5_ and IncFIB(pQil) plasmids harboring *bla*_NDM-1_, respectively. These results indicate that IncFII plasmids harboring *bla*_NDM-5_ and IncFIB(pQil) plasmids harboring *bla*_NDM-1_ have been spreading in K. pneumoniae ST101 and ST147 isolates, respectively, in Myanmar.

**IMPORTANCE** The emergence of carbapenem-resistant K. pneumoniae has become a serious problem in medical settings worldwide. The present study demonstrated that carbapenem-resistant K. pneumoniae strains have been spreading in medical settings in Myanmar. In particular, plasmid genes encoding NDMs and 16S rRNA methylases have been spreading in K. pneumoniae high-risk clones.

## INTRODUCTION

The emergence and spread of carbapenemase-producing *Enterobacteriaceae* (CPE) have become a serious medical problem worldwide (1). Several types of carbapenemases have been detected to date in *Enterobacteriaceae*, with New Delhi metallo-β-lactamase (NDM-type MBL) first being detected in Escherichia coli and Klebsiella pneumoniae isolates obtained from a patient in Sweden in 2008 (2). NDM-type MBLs subsequently spread rapidly worldwide (3), with 40 variants of NDM-type MBLs being detected to date [https://www.ncbi.nlm.nih.gov/pathogens/refgene/#gene_family:(blaNDM)].

NDM-type MBL-producing K. pneumoniae complex isolates have plasmids that carry the *bla*_NDM_ gene (4). Many of these isolates have shown multidrug resistance and have been found to harbor genes, such as *armA* and *rmtB*, encoding 16S rRNA methylases that have been associated with aminoglycoside resistance (4). Plasmids carrying these genes, which have been associated with virulence and antibiotic resistance, belong to various incompatibility (Inc) types.

The present study describes the molecular epidemiology of clinical isolates of K. pneumoniae obtained from patients hospitalized in 10 hospitals in three regions of Myanmar from 2015 to 2017. All of these isolates were carbapenem resistant and produced NDM-type MBLs.

## RESULTS

### Clinical features of carbapenem-resistant K. pneumoniae complex isolates.

The whole genomes of 46 isolates of the K. pneumoniae complex were sequenced using MiSeq. Average nucleotide identity (ANI) and Type (Strain) Genome Sever (TYGS) analyses revealed that 38 were K. pneumoniae subsp. *pneumoniae*, 7 were K. quasipneumoniae subsp. *similipneumoniae*, and 1 was *K. quasipneumoniae* subsp. *quasipneumoniae*. The 46 carbapenem-resistant K. pneumoniae complex strains were isolated from clinical samples obtained from patients hospitalized at 10 hospitals in Myanmar from December 2015 to September 2017. Of the 46 isolates, 30 were from six hospitals in the Yangon region, 14 were from three hospitals in the Mandalay region, and 2 were from one hospital in Kachin State (see Fig. S1 in the supplemental material). The susceptibilities of these isolates to various antibiotics were tested by the microdilution method, according to the guidelines of the Clinical and Laboratory Standards Institute (CLSI) (5). All 46 isolates were resistant to aztreonam (AZT), ceftazidime (CAZ), meropenem (MEM), and tigecycline (TGC); 43 (94%) were resistant to imipenem (IPM); 39 (85%) were resistant to ciprofloxacin (CIP); 27 (59%) were resistant to amikacin (AMK); and 1 (2%) was resistant to colistin (CST) (Table 1).

**TABLE 1:** Drug susceptibility profiles of carbapenem-resistant K. pneumoniae complex isolates in 10 hospitals in Myanmar (*n* = 46)

Antibiotic	Breakpoint for resistance^*a*^ (μg/mL)	% resistant isolates	MIC (μg/mL)		
			Range	MIC_50_	MIC_90_
Amikacin	≥64	50	1 to >1,024	>1,024	>1,024
Aztreonam	≥16	100	16 to >1,024	256	512
Ceftazidime	≥16	100	512 to >1,024	>1,024	>1,024
Ciprofloxacin	≥1	92	0.25 to >1,024	64	256
Colistin	≥4	3	0.0625 to 4	0.25	2
Imipenem	≥4	92	0.5 to 256	8	64
Meropenem	≥4	100	4 to 128	32	128
Tigecycline	≥0.5	100	0.5 to 4	1	2

a The breakpoint for tigecycline was determined according to EUCAST guidelines.

### Drug resistance genes of carbapenem-resistant K. pneumoniae complex isolates.

All 38 isolates of K. pneumoniae subsp. *pneumoniae* harbored *bla*_NDM_ genes, with 22 harboring *bla*_NDM-1_, 1 harboring *bla*_NDM-4_, 11 harboring *bla*_NDM-5_, and 4 harboring *bla*_NDM-7_, as well as *bla*_CTX-M_ genes, including *bla*_CTX-M-15_ or *bla*_CTX-M-14_. Nineteen (50%) isolates also harbored genes encoding 16S rRNA methylases, including *armA* or *rmtB*, making them highly resistant to aminoglycosides. Thirty-three isolates (87%) harbored *aac(6′)-Ib-cr*, which is the most common plasmid-mediated quinolone resistance gene (6). The 26 quinolone-resistant isolates (68%) with MICs of ≥1 μg/mL had point mutations at quinolone resistance-determining regions, including GyrA and ParC (Table 2). A summary of the characteristics of the 8 carbapenem-resistant Klebsiella species, including *K. quasipneumoniae* subsp. *similipneumoniae* and *K. quasipneumoniae* subsp. *quasipneumoniae*, is shown in Table S1. Of them, 6 isolates harbored *bla*_NDM-1_, *bla*_CTX-M-15_, and *armA*, and 2 harbored *bla*_NDM-7_ and *bla*_CTX-M-15_.

**TABLE 2:** Summary of the characteristics of the 38 carbapenem-resistant K. pneumoniae strains, including MLST types and drug resistance genes^*a*^

MLST type	No. of isolates	Hospital(s)	Carbapenemase genes(s)	Extended-spectrum β-lactamase-encoding gene(s)	16S rRNA methylase gene(s)	Aminoglycoside acetyltransferase-encoding gene(s)	Mutation(s) in DNA gyrase	
							GyrA	ParC
ST15	1	A	*bla*_NDM-1_	*bla*_CTX-M-15_, *bla*_SHV-106_	*armA*	*aac(6′)-Ib-cr*, *aac(3)-IId*, *aadA2*, *aadA16*	S83F, D87A	S80I
ST16	2	B (1/2), F (1/2)	*bla*_NDM-1_ (1/2), *bla*_NDM-5_ (1/2)	*bla*_CTX-M-15_, *bla*_SHV-26_, *bla*_SHV-78_, *bla*_SHV-98_	*armA*, *rmtB* (1/2)	*aac(6′)-Ib-cr*, *aadA2*, *aac(6′)*-*Ib3* (1/2), *aac(3)-Ild* (1/2), *aadA16* (1/2)	S83F, D87N	E84K
ST17	2	G	*bla*_NDM-1_	*bla*_CTX-M-14_	—^*b*^	*aac(3)-Iid*, *aac(6′)-Ib-cr*, *aac(6′)-Ib3*	—	—
ST36	1	J	*bla*_NDM-1_	*bla*_CTX-M-15_, *bla*_SHV-11_, *bla*_SHV-13_, *bla*_SHV-70_	*armA*	*aac(3)-Iid*, *aadA16*	—	—
ST42	1	A	*bla*_NDM-4_	*bla*_CTX-M-15_, *bla*_SHV-26_, *bla*_SHV-78_, *bla*_SHV-98_	—	*aac(3)-Iid*, *aac(6′)-Ib-cr*, *aadA16*	S83L, D87Y	S80I
ST101	11	A (8/11),E (2/11),I(1/11)	*bla*_NDM-1_ (3/11), *bla*_NDM-5_ (8/11)	*bla*_CTX-M-15_	*armA*(1/11), *rmtB* (9/11)	*aac(3)-Ild* (8/11), *aac(6′)-Ib-cr* (10/11), *aadA1* (1/11), *aadA2* (8/11), *aadA16* (7/11)	S83Y, D87G (8/11)	S80I (8/11)
ST147	10	A (8/10), C (1/10), G (1/10)	*bla*_NDM-1_ (9/10), *bla*_NDM-5_ (1/10)	*bla*_CTX-M-15_, *bla*_SHV-11_	*rmtB* (1/10)	*aac(3)-Ild* (5/10), *aac(6′)-Ib-cr* (9/10), *aac(6′)-Ib3*, (2/10), *aadA1* (9/10), *aadA2* (4/10), *aadA16* (5/10)	S83I (9/10), S83Y (1/10), D87A (1/10)	S80I
ST273	1	H	*bla*_NDM-7_	*bla*_CTX-M-15_, *bla*_SHV-11_	—	*aac(3)-Ild*, *aac(6′)-Ib-cr*, *aadA16*, *aph(3′)-I*, *aph(6)-Id*	S83I	S80I
ST394	1	C	*bla*_NDM-5_	*bla*_CTX-M-15_	*rmtB*	*aadA2*, *aadA16*	—	—
ST401	1	J	*bla*_NDM-1_	*bla*_CTX-M-15_	*armA*	*aac(6′)-Ib-cr*, *aadA16*	—	—
ST420	1	E	*bla*_NDM-1_	*bla*_CTX-M-15_, *bla*_SHV-75_	*armA*	*aac(3)-IId*, *aadA16*	—	—
ST534	1	A	*bla*_NDM-7_	*bla*_CTX-M-15_, *bla*_SHV-11_, *bla*_SHV-13_, *bla*_SHV-70_, *bla*_SHV-77_, *bla*_SHV-80_	—	*aac(3)*-*Iid*, *aac(6′)*-*Ib-cr*, *aadA16*	S83I	S80I
ST1655	1	G	*bla*_NDM-1_	*bla*_CTX-M-15_, *bla*_SHV-26_, *bla*_SHV-78_, *bla*_SHV-98_	*armA*	*aac(3)-Iid*, *aac(6′)-Ib-cr*, *aadA16*	—	—
ST4029	2	G	*bla*_NDM-1_	*bla*_CTX-M-15_, *bla*_SHV-40_, *bla*_SHV-56_, *bla*_SHV-79_, *bla*_SHV-85_, *bla*_SHV-89_	—	*aac(6′)*-*Ib*-*cr*, *aadA1*, *aac(6′)*-*Ib*	—	—
ST4030	1	G	*bla*_NDM-7_	*bla*_CTX-M-15_, *bla*_SHV-187_	—	*aac(3)-Iid*, *aac(6′)-Ib-cr*, *aadA16*	S83I	S80I
ST5912	1	D	*bla*_NDM-7_	*bla*_CTX-M-15_, *bla*_SHV-26_, *bla*_SHV-78_, *bla*_SHV-98_	—	*aac(6′)-Ib-cr*, *aadA16*	S83F, D87N	E84K

a Numbers in parenthesis indicate that the number of isolates with resistant genes or amino acid mutations per each ST isolate. No parenthesis indicate that all each ST isolate had resistant genes or amino acid mutations.

b — means that the isolate has no mutation, i.e. S83S.

### MLST and phylogenetic analyses of carbapenem-resistant K. pneumoniae complex isolates.

**FIG 1:**
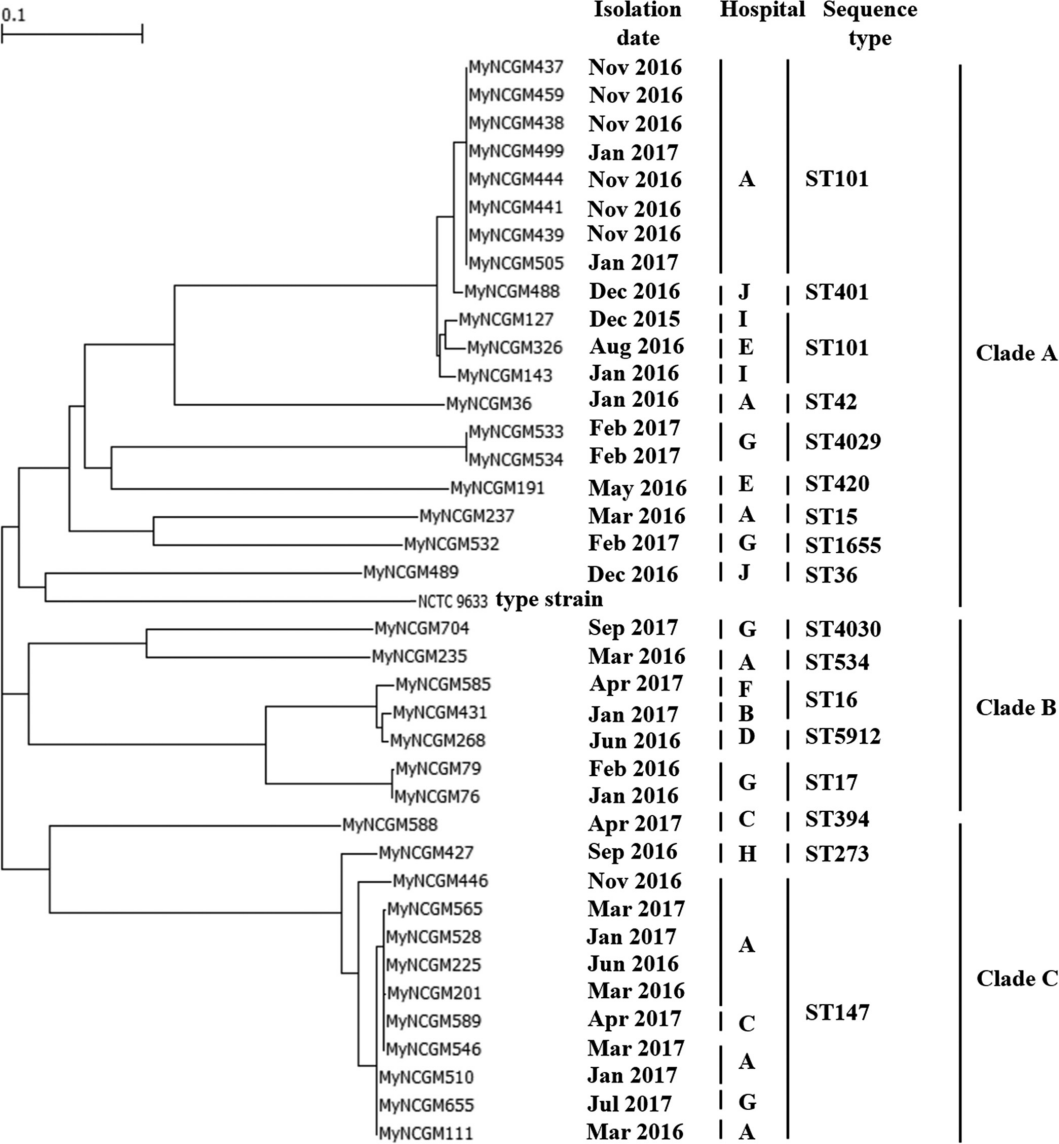
Phylogenetic tree of 38 carbapenem-resistant K. pneumoniae complex isolates obtained from clinical samples at 10 hospitals in Myanmar. The tree was constructed by the maximum likelihood method based on core-genome SNPs.

Multilocus sequence typing (MLST) analysis revealed that 10 isolates (26%) belonged to sequence type 147 (ST147); 11 (29%) belonged to ST101; 2 each (5%) belonged to ST16, ST17, and ST4029; and 1 each (3%) belonged to ST15, ST36, ST42, ST273, ST394, ST401, ST420, ST534, ST1655, ST4030, and ST5912. A phylogenetic tree revealed three clades, designated clades A, B, and C (Fig. 1). Clade A consisted of isolates belonging to ST15, ST36, ST42, ST101, ST401, ST420, ST1655, and ST4029 and the K. pneumoniae reference strain; clade B consisted of isolates belonging to ST16, ST17, ST534, ST4030, and ST5912; and clade C consisted of isolates belonging to ST147, ST273, and ST394. The high-risk clonal lineages ST101 and ST147 belonged to clades A and C, respectively. The other high-risk clonal lineage, ST15, of one isolate belonged to clade A (7). MLST showed that carbapenem-resistant *K. quasipneumoniae* subsp. *similipneumoniae* belonged to ST705, ST1473, ST3590, and ST5967, and *K. quasipneumoniae* subsp. *quasipneumoniae* belonged to ST3866 (Table S1).

As shown in Fig. 1, isolates of the high-risk clonal lineage ST101 in clade A were from hospitals A, E, and I, whereas isolates of the high-risk clonal lineage ST147 in clade C were from hospitals A, C, and G. It is difficult to reveal the relationship between the other STs and hospitals.

Eight isolates belonging to ST101 from hospital A had numbers of single nucleotide polymorphisms (SNPs) ranging from 68 to 126, two ST4029 isolates from hospital G had 68 SNPs, two ST17 isolates from hospital G had 95 SNPs, and five ST147 isolates from hospital A had numbers of SNPs ranging from 93 to 29,684. Of the five ST147 isolates, three isolates (MyNCGM201, MyNCGM225, and MyNCGM528) had numbers of SNPs ranging from 90 to 93.

### Plasmids carrying *bla*_NDM_.

All *bla*_NDM_ genes, including *bla*_NDM-1_, *bla*_NDM-4_, *bla*_NDM-5_, and *bla*_NDM-7_, were located on plasmids ranging in size from 45,321 bp to 176,315 bp. These plasmids belonged to eight types of plasmid incompatibility complexes, including IncC (5 isolates), IncFII (6 isolates), IncFIB(pQil) (9 isolates), IncFIB(pQil)/IncFII(K) (3 isolates), IncFIB(K)/IncFII/IncFII(pKP91) (1 isolate), IncM2 (4 isolates), IncR (1 isolate), and IncX3 (5 isolates) (Table 3). The remaining four plasmids did not belong to any Inc type in *Enterobacteriaceae* (Table 3). The *bla*_NDM-1_ gene was located on IncC, IncFIB(pQil), IncFIB(pQil)/IncFII(K), IncM2, and IncR-type plasmids; *bla*_NDM-4_ was located on IncX3 plasmids; *bla*_NDM-5_ was located on IncFII and IncFIB(K)/IncFII/IncFII(pKP91) plasmids; and *bla*_NDM-7_ was located on IncX3 plasmids (Table 3).

**TABLE 3:** Genetic characterization of carbapenemase resistance plasmids of 38 K. pneumoniae subsp. *pneumoniae* isolates harboring *bla*_NDM_^*a*^

Inc type	No. of isolates	Hospital(s)	MLST type(s)	Plasmid size (bp)	Carbapenemase- and ESBL-encoding gene(s)	Aminoglycoside resistance gene(s)
IncC	5	A (1/5), B (1/5), G (3/5)	ST15 (1/5), ST16 (1/5), ST17 (2/5), ST1655 (1/5)	158,959–176,315	*bla*_NDM-1_	*armA* (3/5), *aac(6′)-Ib-cr*, *aac(6′)-Ib3* (3/5), *aadA2* (1/5)
IncFII	6	A (5/6), C (1/6)	ST101 (4/6), ST147 (1/6), ST394 (1/6)	94,549–94,603	*bla*_NDM-5_	*rmtB*, *aadA2*
IncFIB(pQil)	9	A (7/9), C (1/9), G (1/9)	ST147	51,716–87,316	*bla*_NDM-1_, *bla*_CTX-M-15_	*aac(6′)-Ib-cr*
IncFIB(pQil)/IncFII(K)	3	G (2/3), I (1/3)	ST101 (1/3), ST4029 (2/3)	119,263–126,228	*bla*_NDM-1_, *bla*_CTX-M-15_	*aac(6′)-Ib-cr*, *aac(6′)-Ib*, *aadA1*
IncFIB(K)/IncFII/IncFII(pKP91)	1	A	ST101	199,295	*bla*_NDM-5_, *bla*_CTX-M-15_	*rmtB*, *aac(6′)-Ib-cr*, *aac(3)-Iid*, *aadA2*, *aadA16*
IncM2	4	E (1/4), I (1/4), J (2/4)	ST36 (1/4), ST101 (1/4), ST401 (1/4), ST420 (1/4)	80,663–80,798	*bla*_NDM-1_	*armA*
IncR	1	E	ST101	67,399	*bla*_NDM-1_	*rmtB*, *aac(6′)-Ib-cr*, *aadA16*
IncX3	5	A (2/5), D (1/5), H (1/5), G (1/5)	ST42 (1/5), ST273 (1/5), ST534 (1/5), ST4030 (1/5), ST5912 (1/5)	45,321–46,161	*bla*_NDM-4_ (1/5), *bla*_NDM-7_ (4/5)	—
—^*b*^	4	A (3/4), F (1/4)	ST16 (1/4), ST101 (3/4)	10,494–122,000	*bla*_NDM-5_	*aadA2* (3/4), *aac(6′)-Ib-cr* (1/4), *aadA16* (1/4)

a Numbers in parenthesis indicate that the number of isolates with resistant genes or amino acid mutations per each ST isolate. No parenthesis indicate that all each ST isolate had resistant genes or amino acid mutations.

b — means that the isolate has no mutation, i.e. S83S.

Of the 38 plasmids carrying *bla*_NDM_, 15 (39%) harbored genes encoding both NDMs and 16S rRNA methylases. These 15 plasmids included 7 that harbored *armA* on IncC- or IncM2-type plasmids and 8 that harbored *rmtB* on IncFII-, IncFIB(K)/IncFII/IncFII(pKP91)-, or IncR-type plasmids (Table 3).

The IncFIB(pQil)-type plasmids carrying *bla*_NDM_ were detected in isolates from three regions in Myanmar, Kachin, Mandalay, and Yangon, whereas the IncC-type, IncFIB(pQil)/IncFII(K), IncM2, and IncX3-type plasmids carrying *bla*_NDM_ were detected in isolates from urban areas, including the Mandalay and Yangon regions, in Myanmar (Table 3 and Fig. S1).

### Genetic environments surrounding *bla*_NDM_ and 16S rRNA methylases.

**FIG 2:**
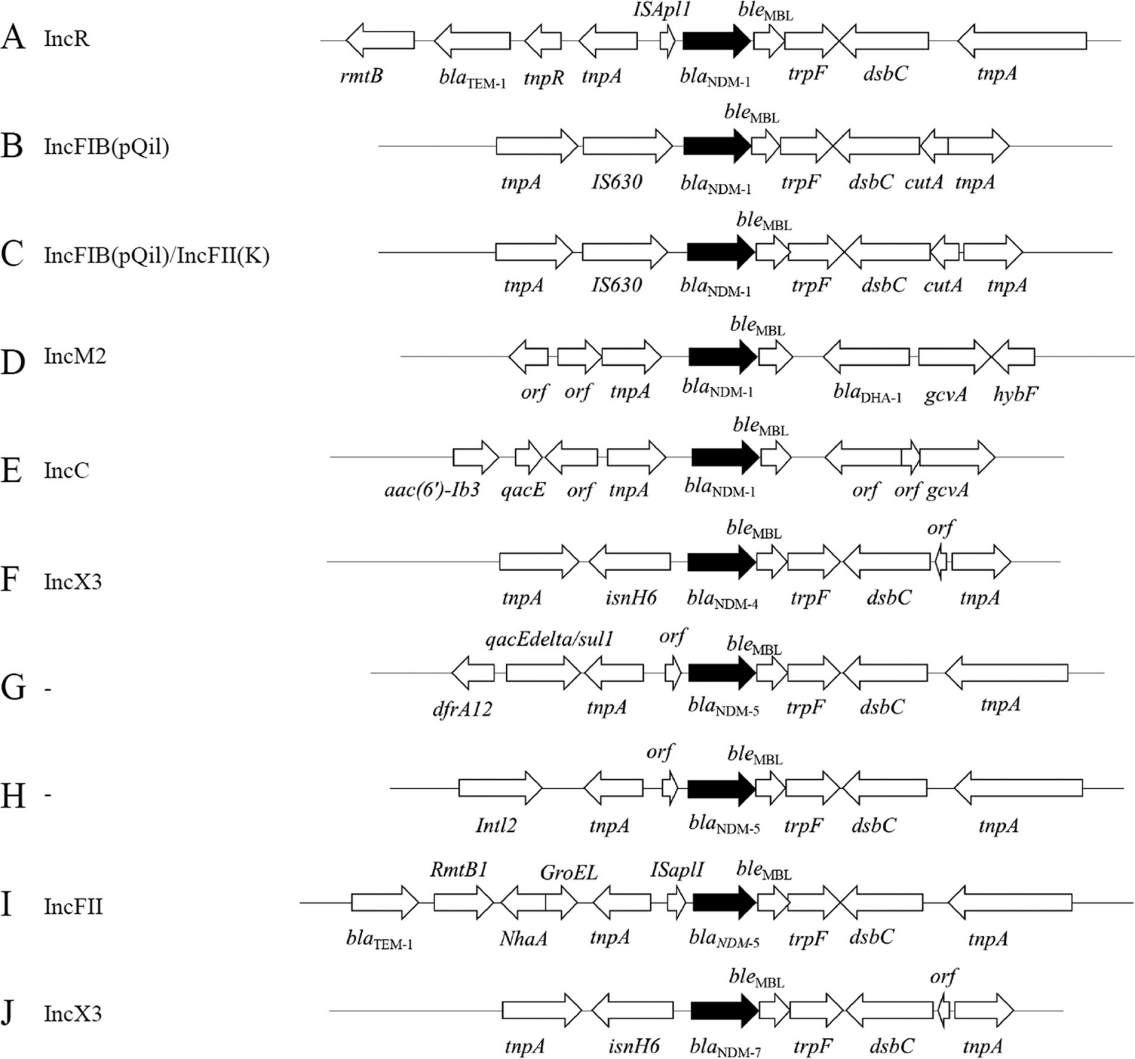
Genomic environments of *bla*_NDM_ in K. pneumoniae complex strains isolated from various clinical samples obtained at 10 hospitals in Myanmar.

Assessment of the genomic environments surrounding *bla*_NDM_ revealed 10 types of genetic structures, including *bla*_NDM-1_ (Fig. 2A to E), *bla*_NDM-4_ (Fig. 2F), *bla*_NDM-5_ (Fig. 2G to I), and *bla*_NDM-7_ (Fig. 2J).

The genetic structure surrounding *bla*_NDM-1_ could be divided into five types (Fig. 2A to E). The structure of type A was *rmtB-bla*_TEM-1_-*tnpR*-*tnpA-*IS*Apll-bla*_NDM-1_*-ble*_MBL_-*trpF-dsbC-tnpA*. The structure *tnpA-*IS*Apll-bla*_NDM-1_*-ble*_MBL_-*trpF-dsbC-tnpA* was identical to those of plasmids in other types of *Enterobacteriaceae*, including Escherichia coli pC06114_1 (GenBank accession no. CP016035) detected in 2015 in Germany and K. pneumoniae pM941-NDM5 (GenBank accession no. AP023454) detected in 2018 in Myanmar. The structure of types B and C was *tnpA-*IS*630*-*bla*_NDM-1_*-ble*_MBL_-*trpF*-*dsbC*-*cutA*-*tnpA*, which was identical to the structures of plasmids of K. pneumoniae AATZP (GenBank accession no. CP014757) detected in 2014 in the United States; K. pneumoniae K66-45 (GenBank accession no. CP020902) detected in 2010 in Norway; and K. pneumoniae C435, C069, and C070 (GenBank accession no. LC521845, LC613144, and LC521839, respectively) detected in Thailand in 2016. The structure of type D was *orf-orf*-*tnpA-bla*_NDM-1_*-ble*_MBL_-*bla*_DHA-1_-*gcvA-hybF*, which was identical to the structures of plasmids of E. coli Es_ST2350_SE1 (GenBank accession no. CP031322), first detected in 2018 in the United Kingdom, and K. pneumoniae 3347689I (GenBank accession no. CP071086), first detected in 2020 in Switzerland. The structure of type E was *aac(6′)-Ib3-qacE-orf*-*tnpA*-*bla*_NDM-1_*-ble*_MBL_-*orf-orf*-*gcvA*, which was identical to the structure of a plasmid in E. coli Carbapenemase (NDM-1)_IncA/C2 (GenBank accession no. CP050162), first detected in 2012 in Hong Kong.

The genetic structure surrounding *bla*_NDM-4_ was *tnpA-isnH6-bla*_NDM-4_-*ble*_MBL_*-trpF-dsbC-orf-tnpA* (Fig. 2F), which was identical to those of plasmids of E. coli M2-16 (GenBank accession no. AP018146) in 2015 in Myanmar and E. coli TUM18530 (GenBank accession no. AP023194) in 2018 in Japan.

Three genetic structures were observed to surround *bla*_NDM-5_ (Fig. 2G to I). The structure of type G was *dfrA12-qacEΔ/sul1-tnpA-orf-bla*_NDM-5_-*ble*_MBL_-*trpF-dsbC-tnpA*, which was identical to those of plasmids of E. coli isolated from 2013 to 2019 in China, Malawi, Myanmar, South Korea, Thailand, and the United States. The structure of type H was *Intl2*-*tnpA*-*orf*-*bla*_NDM-5_*-ble*_MBL_-*trpF*-*dsbC*-*tnpA*, which was identical to that of a plasmid of Enterobacter hormaechei, p388, isolated in 2017 in the United States (GenBank accession no. CP021168). The structure of type I was *bla*_TEM-1_*-rmtB1-nhaA-groEL-tnpA-*IS*Apll-bla*_NDM-5_*-ble*_MBL_*-trpF-dsbC-tnpA*, which was identical to those of E. coli plasmids pM214_FII and pM105_mF (GenBank accession no. AP018144 and AP018137, respectively), isolated in 2015 in Myanmar.

The genetic structure surrounding *bla*_NDM-7_ (Fig. 2J), *tnpA-isnH6-bla*_NDM-7_*-ble*_MBL_-*trpF-dsbC-orf-tnpA*, was similar to that surrounding *bla*_NDM-4_, with the latter being identical to those of plasmids of E. coli M2-16 (GenBank accession no. AP018146) in 2015 in Myanmar and E. coli TUM18530 (GenBank accession no. AP023194) in 2018 in Japan.

**FIG 3:**
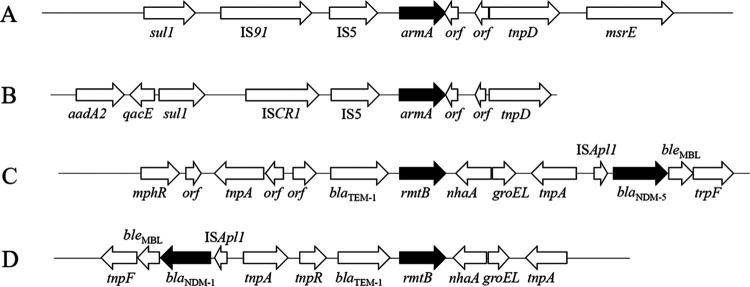
Genomic environments of *armA* and *rmtB* in K. pneumoniae complex strains isolated from various clinical samples obtained at 10 hospitals in Myanmar.

The structures of the genomic environments surrounding *armA* and *rmtB* are shown in Fig. 3. The structure surrounding *armA* of type A was detected in four of seven isolates and was identical to those in E. coli isolated from 2003 to 2018 in China, Hong Kong, India, Norway, and Poland (GenBank accession no. CP072463, HQ451074, CP030858, CP020902, and CP058363, respectively). The structure surrounding *armA* of type B was detected in three of seven isolates and was identical to those in K. pneumoniae isolated in Oman, Japan, and South Africa (GenBank accession no. JX988621, AB759690, and CP023488, respectively). The structure surrounding *rmtB* of type C was detected in 11 of 12 isolates and was identical to those in a strain of K. pneumoniae isolated in 2018 in the Czech Republic (GenBank accession no. CP050367) and strains of E. coli isolated from 2012 to 2019 in India, Italy, and Switzerland (GenBank accession no. CP033159, MN007141, and CP048368, respectively). The structure surrounding *rmtB* of type D was detected in 1 of the 12 isolates.

Of the 38 isolates, 7 harbored both *bla*_NDM-1_ and *armA* on the same plasmids, including four IncM2 and three IncC plasmids; 6 harbored both *bla*_NDM-5_ and *rmtB* on the same plasmids, including plasmid type IncFII or IncFIB(K)/IncFII/IncFII (pKP91); and 1 harbored both *bla*_NDM-1_ and *rmtB* on the same plasmid belonging to IncR.

**FIG 4:**
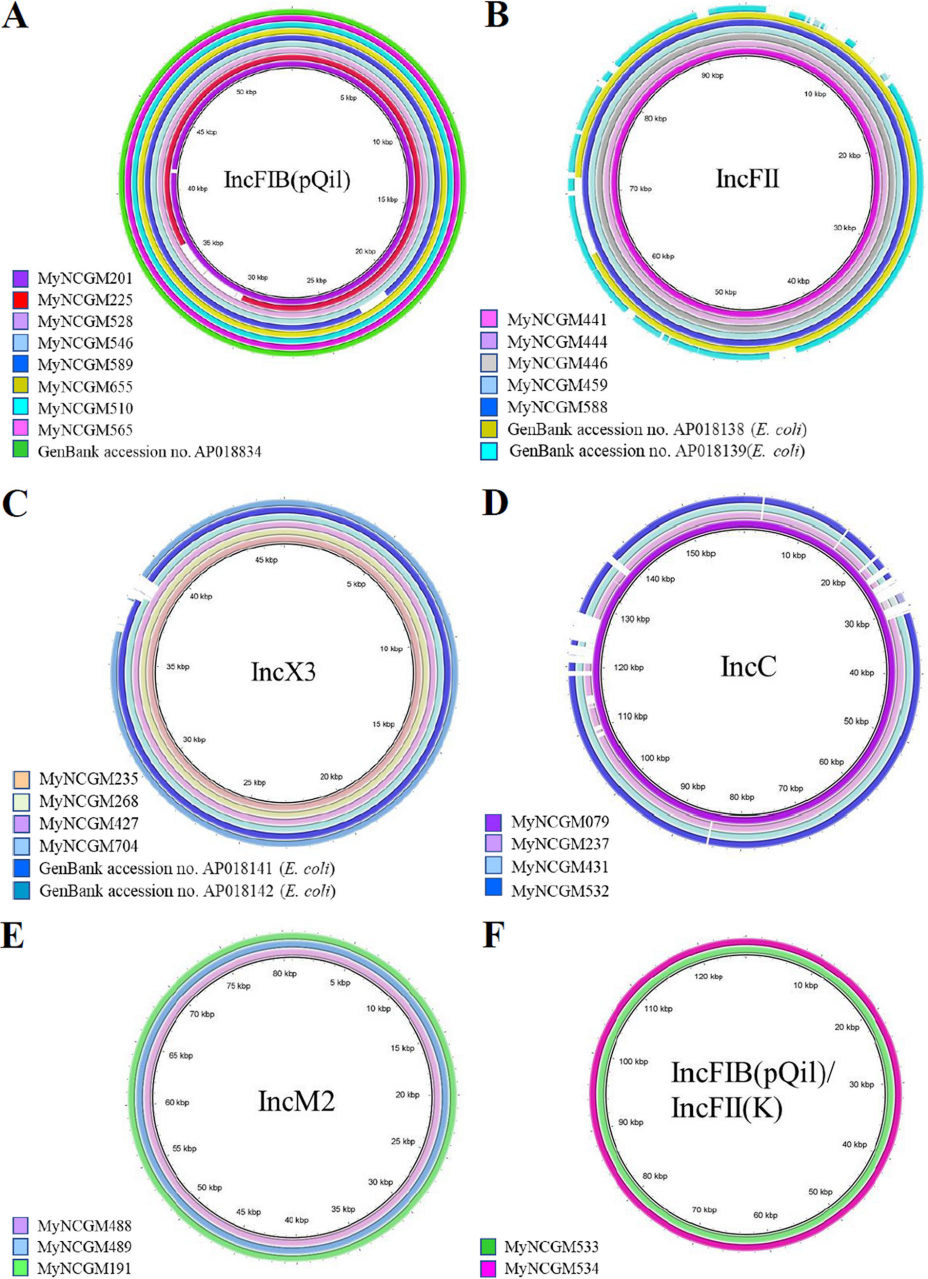
Comparison of the plasmid sequences of IncFIB(pQil), IncFII, IncX3, IncC, IncM2, and IncFIB(pQil)/IncFII(K). The images were generated using BLAST Ring Image Generator software (https://sourceforge.net/projects/brig/files/BRIG-0.95-dist.zip/download). Plasmid sequences belonging to each Inc type were compared with plasmids of MyNCGM111 for IncFIB(pQil) (A), MyNCGM439 for IncFII (B), MyNCGM036 for IncX3 (C), MyNCGM076 for IncC (D), MyNCGM127 for IncM2 (E), and MyNCGM143 for IncFIB(pQil)/IncFII(K) (F).

The plasmid structures belonging to IncFIB(pQil), IncFII, IncX3, IncC, IncM2, and IncFIB(pQil)/IncFII(K) are compared in Fig. 4. Five of nine plasmids belonging to IncFIB(pQil) had structures identical to that of a plasmid in K. pneumoniae in 2015 in Myanmar (GenBank accession no. AP018834). All six plasmids belonging to IncFII had structures 92% identical to that of a plasmid in E. coli isolated in 2015 in Myanmar (GenBank accession no. AP018138). Six of seven plasmids belonging to IncX3 had structures 97% identical to that of a plasmid in E. coli isolated in 2015 in Myanmar (GenBank accession no. AP018141). On the other hand, *K. quasipneumoniae* subsp. *similipneumoniae* harbored IncX3, IncC, or IncM2 plasmids, and *K. quasipneumoniae* subsp. *quasipneumoniae* harbored an IncX3 plasmid (Fig. 4).

## DISCUSSION

The present study suggests that K. pneumoniae ST101 isolates harboring *bla*_NDM-5_ on IncFII plasmids and ST147 isolates harboring *bla*_NDM-1_ on IncFIB(pQil) plasmids have spread in three regions in Myanmar in recent years. IncFII plasmids harboring *bla*_NDM-5_ in ST101 isolates and IncFIB(pQil) plasmids harboring *bla*_NDM-1_ in ST147 isolates seem to be horizontally spreading in hospital A. IncFII and IncFIB(pQil) plasmids harboring *bla*_NDM_ were detected in E. coli ST354 and K. pneumoniae ST147 strains isolated in Myanmar (8), the United States (GenBank accession no. CP014757), Norway (GenBank accession no. CP020902), and Thailand (GenBank accession no. LC521839).

IncX3 plasmids harboring *bla*_NDM_s will be spreading among *Enterobacteriaceae*, including *Citrobacter* sp., Enterobacter sp., E. coli, and K. pneumoniae subspecies in Myanmar. In this study, we revealed that K. pneumoniae subsp. *pneumoniae*, *K. quasipneumoniae* subsp. *similipneumoniae*, and *K. quasipneumoniae* subsp. *quasipneumoniae* had IncX3 plasmids harboring *bla*_NDM-4_ or *bla*_NDM-7_ (Table 3; see also Table S1 in the supplemental material). Sugawara et al. reported that Citrobacter amalonaticus, Citrobacter freundii, Enterobacter asburiae, Enterobacter xiangfangensis, E. coli, Klebsiella pneumoniae, Klebsiella quasipneumoniae, Leclercia adecarboxylata, and Lelliottia nimipressuralis harbored IncX3 plasmids harboring *bla*_NDM-4_, *bla*_NDM-5_, or *bla*_NDM-7_ in Myanmar (8). Another study showed that IncX3 plasmids harboring *bla*_NDM-5_ have spread in K. pneumoniae isolates in China (9).

Carbapenem-resistant *Enterobacteriaceae* are a significant public health concern in Myanmar (8). K. pneumoniae ST101 and ST147 isolates producing NDM-1 caused outbreaks in Spain (10), and ST101 isolates producing extended-spectrum β-lactamases (ESBLs) were also reported in Tanzania (11). Previous studies in Myanmar revealed that K. pneumoniae ST101 and ST147 strains were detected in samples from patients in medical settings and environments, including foodstuff in the Yangon region (12, 13). In addition to ST101 and ST147, other high-risk clones, ST11, ST15, ST14, and ST48, were reported in Myanmar (14), Bangladesh (7), Saudi Arabia (15), and China (16), respectively.

The results of SNP analysis of closely related isolates (Fig. 1) suggested that eight ST101 isolates from hospital A, two ST4029 isolates from hospital G, two ST17 isolates from hospital G, and three ST147 isolates from hospital A represented outbreaks.

Strains of *Enterobacteriaceae* containing plasmids carrying genes encoding NDMs and 16S rRNA methylases, making these bacteria resistant to carbapenems and aminoglycosides, will likely spread throughout medical settings in Myanmar. Isolates of the Enterobacter cloacae complex coproducing NDM-1/4 and ArmA/RmtC/RmtE have been detected in five regions in Myanmar (17), and other species of *Enterobacteriaceae*, including E. coli and Citrobacter freundii, resistant to carbapenems and aminoglycosides and producing NDM-1/4/5 and ArmA/RmtB/RmtC/RmtE have been detected in environments as well as medical settings in Yangon, Myanmar (13). These findings emphasize the need to monitor *Enterobacteriaceae* in Myanmar for the presence of plasmid-borne genes encoding carbapenemases and 16S rRNA methylases.

In conclusion, this is the first report describing the molecular epidemiology of carbapenem-resistant K. pneumoniae isolates in medical settings in three regions of Myanmar. The incidence of multidrug-resistant (MDR) K. pneumoniae clinical isolates in hospitals differed regionally, being 57.9% in the Yangon region (8) but 39.5% in the three regions included in the present study. Epidemiological surveillance is required to prevent the emergence and spread in Myanmar of MDR *Enterobacteriaceae* harboring genes encoding enzymes associated with drug resistance.

## MATERIALS AND METHODS

### Bacterial strains.

Forty-six clinical isolates of the carbapenemase-resistant K. pneumoniae complex, defined as strains showing resistance to imipenem or meropenem (MIC, ≥4 μg/mL), were obtained between December 2015 and September 2017 from patients treated at 10 hospitals in Myanmar. Of these 46 isolates, 20, 1, 2, 1, 4, 1, 8, 5, 2, and 2 were from hospitals A through J, respectively. Bacteria were identified using the Vitek 2 system (bioMérieux, Marcy l’Etoile, France), with identities confirmed by sequencing of 16S rRNA. Of the 46 isolates, 22 were from blood, 10 were from tracheal aspirates and sputum, 7 were from pus and wounds, and 7 were from urine. As the situation in Myanmar has become increasingly uncertain in recent months, it is difficult to update the clinical information on the 46 K. pneumoniae isolates tested.

### Drug susceptibility testing.

Drug susceptibility was tested according to the guidelines of the Clinical and Laboratory Standards Institute (CLSI) (5). The ranges of antibiotic concentrations tested were 0.5 to 1,024 μg/mL amikacin (AMK), 0.5 to 1,024 μg/mL aztreonam (AZT), 0.5 to 1,024 μg/mL ceftazidime (CAZ), 0.25 to 1,024 μg/mL ciprofloxacin (CIP), 0.0625 to 8 μg/mL colistin (CST), 0.5 to 1,024 μg/mL imipenem (IPM), 0.5 to 1,024 μg/mL meropenem (MEM), and 0.5 to 1,024 μg/mL tigecycline (TGC) (Table 1). The MICs of each antimicrobial agent were determined by broth microdilution methods using Mueller-Hinton broth and 96-well microtiter plates (Kohjin Bio Co., Ltd., Saitama, Japan).

### Whole-genome sequencing and genomic analysis.

Genomic DNAs of the 46 isolates were extracted using DNeasy blood and tissue kits (Qiagen, Tokyo, Japan) or 20-gauge genomic tips (Qiagen), and their complete genomes were sequenced using the MiSeq platform (Illumina, San Diego, CA) and MinION (Oxford Nanopore Technologies, Oxford, United Kingdom). Raw reads of each isolate were assembled using CLC Genomic Workbench version 10.0.1 (CLC Bio, Aarhus, Denmark). Species identities of these isolates were determined using an ANI calculator (18) or the Type (Strain) Genome Sever (TYGS) (https://tygs.dsmz.de). The sequences of drug resistance genes were determined using ResFinder 4.1, and plasmids were typed using Plasmid finder 2.1, both from the Center for Genomic Epidemiology (CGE) (https://www.genomicepidemiology.org/). The sequences of plasmids were annotated using the DDBJ Fast annotation and submission tool (https://dfast.ddbj.nig.ac.jp). Fluoroquinolone resistance has been associated with mutations in the quinolone resistance-determining region, which includes the *gyrA* and *parC* genes that encode DNA gyrase and topoisomerase IV, respectively. The *gyrA* and *parC* genes were detected *in silico* using CLC Genomics Workbench v.11.0.1 (CLC Bio, Denmark) (19). Comparative analysis of plasmid sequences surrounding *bla*_NDM_ was performed using BLAST and visualized using *in silico* molecular cloning (*In Silico* Biology Inc., Kanagawa, Japan). Imaging of plasmid similarity was performed using the BLAST Ring Image Generator (https://sourceforge.net/projects/brig/files/BRIG-0.95-dist.zip/download).

### MLST and phylogenetic analyses.

Multilocus sequence typing (MLST) was performed according to protocols of the MLST databases (https://bigsdb.pasteur.fr/). Phylogenetic trees were constructed using kSNP3.1 software (https://sourceforge.net/projects/ksnp/files/) (20) and visualized using FigTree v.1.4.4 (https://github.com/rambaut/figtree/releases). The type strain K. pneumoniae NCTC 9633 was used as the reference strain.

### Accession number(s).

The whole-genome sequences of all 46 isolates have been deposited in GenBank under accession no. DRA009233.

## References

[1] Bush K. 2001. New beta-lactamases in Gram-negative bacteria: diversity and impact on the selection of antimicrobial therapy. Clin Infect Dis 32:1085–1089. doi:10.1086/319610. 11264037

[2] Lee CR, Lee JH, Park KS, Kim YB, Jeong BC, Lee SH. 2016. Global dissemination of carbapenemase-producing *Klebsiella pneumoniae*: epidemiology, genetic context, treatment options, and detection methods. Front Microbiol 7:895. doi:10.3389/fmicb.2016.00895. 27379038 PMC4904035

[3] Nordmann P, Naas T, Poirel L. 2011. Global spread of carbapenemase-producing *Enterobacteriaceae*. Emerg Infect Dis 17:1791–1798. doi:10.3201/eid1710.110655. 22000347 PMC3310682

[4] Pitout JD, Nordmann P, Poirel L. 2015. Carbapenemase-producing *Klebsiella pneumoniae*, a key pathogen set for global nosocomial dominance. Antimicrob Agents Chemother 59:5873–5884. doi:10.1128/AAC.01019-15. 26169401 PMC4576115

[5] Clinical and Laboratory Standards Institute. 2019. Performance standards for antimicrobial susceptibility testing, 29th ed. CLSI supplement M100. Clinical and Laboratory Standards Institute, Wayne, PA.

[6] Aung MS, Win NC, San N, Hlaing MS, Myint YY, Thu PP, Aung MT, Yaa KT, Maw WW, Urushibara N, Kobayashi N. 2021. Prevalence of extended-spectrum β-lactamase/carbapenemase genes and quinolone-resistance determinants in *Klebsiella pneumoniae* clinical isolates from respiratory infections in Myanmar. Microb Drug Resist 27:36–43. doi:10.1089/mdr.2019.0490. 32522093

[7] Farzana R, Jones LS, Barratt A, Rahman MA, Sands K, Portal E, Boostrom I, Espina L, Pervin M, Uddin A, Walsh TR. 2020. Emergence of mobile colistin resistance (*mcr-8*) in a highly successful *Klebsiella pneumoniae* sequence type 15 clone from clinical infections in Bangladesh. mSphere 5:e00023-20. doi:10.1128/mSphere.00023-20. 32161143 PMC7067589

[8] Sugawara Y, Akeda Y, Hagiya H, Sakamoto N, Takeuchi D, Shanmugakani RK, Motooka D, Nishi I, Zin KN, Aye MM, Myint T, Tomono K, Hamada S. 2019. Spreading patterns of NDM-producing *Enterobacteriaceae* in clinical and environmental settings in Yangon, Myanmar. Antimicrob Agents Chemother 63:e01924-18. doi:10.1128/AAC.01924-18. 30530602 PMC6395922

[9] Zhu W, Wang X, Qin J, Liang W, Shen Z. 2020. Dissemination and stability of the *bla*_NDM-5_-carrying IncX3-type plasmid among multiclonal *Klebsiella pneumoniae* isolates. mSphere 5:e00917-20. doi:10.1128/mSphere.00917-20. PMC7643832 33148824

[10] Perez-Vazquez M, Sola Campoy PJ, Ortega A, Bautista V, Monzon S, Ruiz-Carrascoso G, Mingorance J, Gonzalez-Barbera EM, Gimeno C, Aracil B, Saez D, Lara N, Fernandez S, Gonzalez-Lopez JJ, Campos J, Kingsley RA, Dougan G, Oteo-Iglesias J, Spanish NDM Study Group. 2019. Emergence of NDM-producing *Klebsiella pneumoniae* and *Escherichia coli* in Spain: phylogeny, resistome, virulence and plasmids encoding *bla*_NDM_-like genes as determined by WGS. J Antimicrob Chemother 74:3489–3496. doi:10.1093/jac/dkz366. 31504589

[11] Büdel T, Kuenzli E, Clément M, Bernasconi OJ, Fehr J, Mohammed AH, Hassan NK, Zinsstag J, Hatz C, Endimiani A. 2019. Polyclonal gut colonization with extended-spectrum cephalosporin- and/or colistin-resistant *Enterobacteriaceae*: a normal status for hotel employees on the island of Zanzibar, Tanzania. J Antimicrob Chemother 74:2880–2890. doi:10.1093/jac/dkz296. 31361004

[12] Sugawara Y, Akeda Y, Hagiya H, Zin KN, Aye MM, Takeuchi D, Matsumoto Y, Motooka D, Nishi I, Tomono K, Hamada S. 2021. Characterization of *bla*_NDM-5_-harbouring *Klebsiella pneumoniae* sequence type 11 international high-risk clones isolated from clinical samples in Yangon General Hospital, a tertiary-care hospital in Myanmar. J Med Microbiol 70:1348. doi:10.1099/jmm.0.001348. 34038339

[13] Sugawara Y, Hagiya H, Akeda Y, Aye MM, Myo Win HP, Sakamoto N, Shanmugakani RK, Takeuchi D, Nishi I, Ueda A, Htun MM, Tomono K, Hamada S. 2019. Dissemination of carbapenemase-producing *Enterobacteriaceae* harbouring *bla*_NDM_ or *bla*_IMI_ in local market foods of Yangon, Myanmar. Sci Rep 9:14455. doi:10.1038/s41598-019-51002-5. 31595007 PMC6783431

[14] Sakamoto N, Akeda Y, Sugawara Y, Takeuchi D, Motooka D, Yamamoto N, Laolerd W, Santanirand P, Hamada S. 2018. Genomic characterization of carbapenemase-producing *Klebsiella pneumoniae* with chromosomally carried *bla*_NDM-1_. Antimicrob Agents Chemother 62:e01520-18. doi:10.1128/AAC.01520-18. 30323033 PMC6256778

[15] Alghoribi MF, Alqurashi M, Okdah L, Alalwan B, AlHebaishi YS, Almalki A, Alzayer MA, Alswaji AA, Doumith M, Barry M. 2021. Successful treatment of infective endocarditis due to pandrug-resistant *Klebsiella pneumoniae* with ceftazidime-avibactam and aztreonam. Sci Rep 11:9684. doi:10.1038/s41598-021-89255-8. 33958683 PMC8102575

[16] Tian D, Wang B, Zhang H, Pan F, Wang C, Shi Y, Sun Y. 2020. Dissemination of the *bla*_NDM-5_ gene via IncX3-type plasmid among *Enterobacteriaceae* in children. mSphere 5:e00699-19. doi:10.1128/mSphere.00699-19. PMC6952193 31915216

[17] Oshiro S, Tada T, Watanabe S, Tohya M, Hishinuma T, Uchida H, Kuwahara-Arai K, Mya S, Zan KN, Kirikae T, Tin HH. 2020. Emergence and spread of carbapenem-resistant and aminoglycoside-panresistant *Enterobacter cloacae* complex isolates coproducing NDM-type metallo-β-lactamase and 16S rRNA methylase in Myanmar. mSphere 5:e00054-20. doi:10.1128/mSphere.00054-20. 32161144 PMC7067590

[18] Yoon SH, Ha SM, Lim J, Kwon S, Chun J. 2017. A large-scale evaluation of algorithms to calculate average nucleotide identity. Antonie Van Leeuwenhoek 110:1281–1286. doi:10.1007/s10482-017-0844-4. 28204908

[19] Deguchi T, Fukuoka A, Yasuda M, Nakano M, Ozeki S, Kanematsu E, Nishino Y, Ishihara S, Ban Y, Kawada Y. 1997. Alterations in the GyrA subunit of DNA gyrase and the ParC subunit of topoisomerase IV in quinolone-resistant clinical isolates of *Klebsiella pneumoniae*. Antimicrob Agents Chemother 41:699–701. doi:10.1128/AAC.41.3.699. 9056017 PMC163775

[20] Gardner SN, Slezak T, Hall BG. 2015. kSNP3.0: SNP detection and phylogenetic analysis of genomes without genome alignment or reference genome. Bioinformatics 31:2877–2878. doi:10.1093/bioinformatics/btv271. 25913206

