# Supplementary material for: Spread of Carbapenem-Resistant Klebsiella pneumoniae Clinical Isolates Producing NDM-Type Metallo-β-Lactamase in Myanmar
Source: Microbiol Spectr. 2022 Jun 28;10(4):e00673-22. doi: 10.1128/spectrum.00673-22 (PMC9431462; doi:10.1128/spectrum.00673-22)
Supplement: Supplemental file 1 — Supplemental material. Download spectrum.00673-22-s0001.pdf, PDF file, 1.3 MB [file spectrum.00673-22-s0001.pdf]

Table S1 Summary of the characteristics of the 8 carbapenem-resistant *K. quasipneumoniae subsp. similipneumoniae* and *K. quasipneumoniae subsp. quasipneumoniae*, including MLSTs and drug resistance genes

MLST	Plasmid type	Number of isolates	Hospitals	Carbapenemase genes(s)	Extended spectrum β-lactamase encoding gene(s)	16S rRNA methylase gene(s)	Aminoglycoside acetyltransferase-encoding gene(s)	Mutation(s) in DNA gyrase	
								GyrA	ParC
ST705	IncM2	2	E	*bla*$_{\text{NDM-1}}$	*bla*$_{\text{CTX-M-15}}$	*armA*	*aac(3)-IId, aadA16*	-	-
ST1473	IncC	3	H	*bla*$_{\text{NDM-1}}$	*bla*$_{\text{CTX-M-15}}$	*armA*	*aac(6')-Ib-cr*	-	-
ST3590	IncX3	1	A	*bla*$_{\text{NDM-7}}$	*bla*$_{\text{CTX-M-15}}$	-	*aac(6')-Ib-cr*	-	-
ST3866	IncX3	1	H	*bla*$_{\text{NDM-7}}$	*bla*$_{\text{CTX-M-15}}$	-	*aac(3)-IIa, aac(6')-Ib-cr, aadA2*	-	-
ST5967	IncFIB(pNDM-Mar)/ IncHI1B(pNDM-MAR)	1	G	*bla*$_{\text{NDM-1}}$	*bla*$_{\text{CTX-M-15}}$	*armA*	*aac(3)-IId, aac(6')-Ib-cr, aadA2, aadA16*	-	-

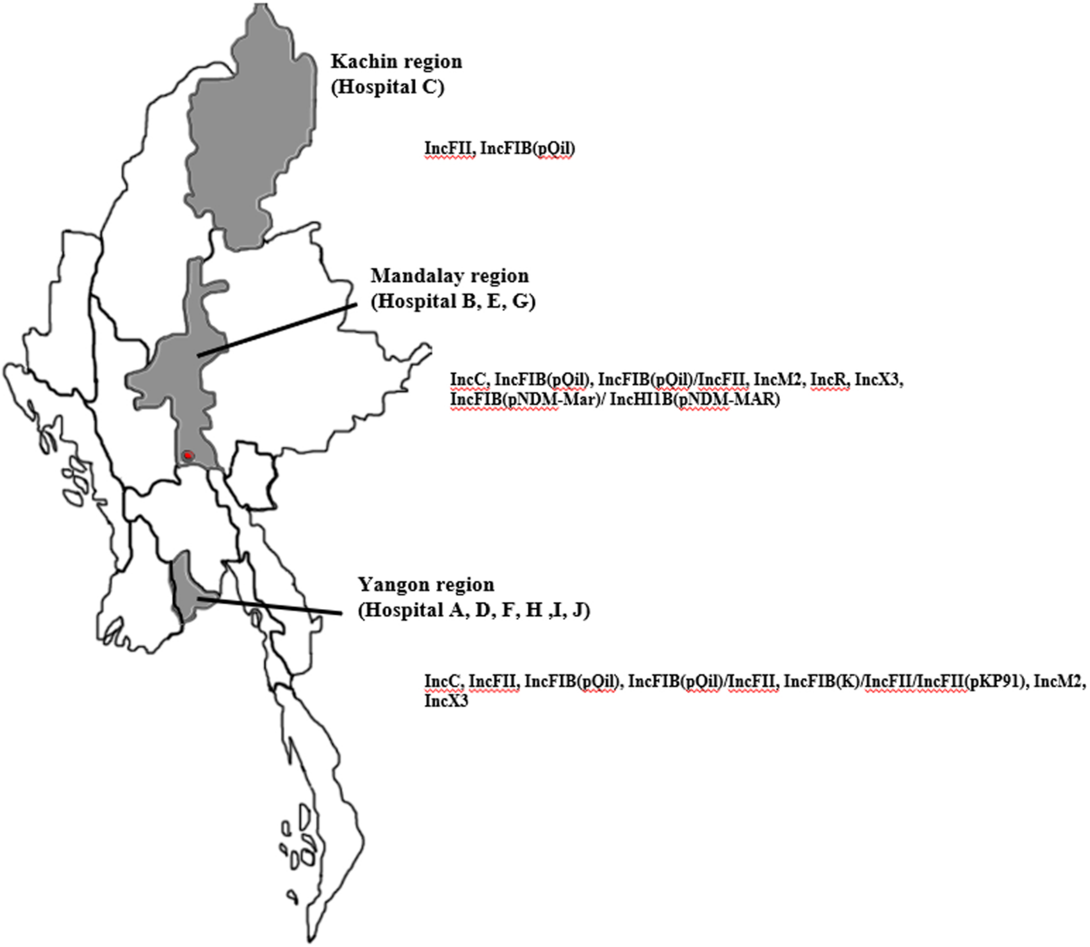

Figure S1

Relation between geographical locations of the 10 hospitals in Myanmar and plasmid Inc types from the 46 carbapenem-resistant *K. pneumoniae* complex isolates. IncFIB(pQil) are spreading in three regions of Myanmar.
